# CADD: predicting the deleteriousness of variants throughout the human genome

**DOI:** 10.1093/nar/gky1016

**Published:** 2018-10-29

**Authors:** Philipp Rentzsch, Daniela Witten, Gregory M Cooper, Jay Shendure, Martin Kircher

**Affiliations:** 1 Berlin Institute of Health (BIH), 10178 Berlin, Germany; 2 Charité - Universitätsmedizin Berlin, 10117 Berlin, Germany; 3 Department of Statistics and Biostatistics, University of Washington, Seattle, WA 98195, USA; 4 HudsonAlpha Institute for Biotechnology, Huntsville, AL 35806, USA; 5 Department of Genome Sciences, University of Washington, Seattle, WA 98195, USA; 6 Brotman Baty Institute for Precision Medicine, Seattle, WA 98195, USA

## Abstract

Combined Annotation-Dependent Depletion (CADD) is a widely used measure of variant deleteriousness that can effectively prioritize causal variants in genetic analyses, particularly highly penetrant contributors to severe Mendelian disorders. CADD is an integrative annotation built from more than 60 genomic features, and can score human single nucleotide variants and short insertion and deletions anywhere in the reference assembly. CADD uses a machine learning model trained on a binary distinction between simulated *de novo* variants and variants that have arisen and become fixed in human populations since the split between humans and chimpanzees; the former are free of selective pressure and may thus include both neutral and deleterious alleles, while the latter are overwhelmingly neutral (or, at most, weakly deleterious) by virtue of having survived millions of years of purifying selection. Here we review the latest updates to CADD, including the most recent version, 1.4, which supports the human genome build GRCh38. We also present updates to our website that include simplified variant lookup, extended documentation, an Application Program Interface and improved mechanisms for integrating CADD scores into other tools or applications. CADD scores, software and documentation are available at https://cadd.gs.washington.edu.

## INTRODUCTION

Human genome sequencing is now routine, and facilitates the ascertainment of millions of genetic variants within individuals, and hundreds of millions of variants across populations (1). However, the interpretation of genetic variants remains an enormous challenge, and it is clear that the further development of methods to prioritize variants that substantially impact human phenotypes is essential to maximize the utility of sequencing data. Genetic strategies to identify such variants include genome-wide association, linkage and family or trio studies. However, the resolution of purely genetic strategies is limited by statistical power and other factors (2). Complementary methods to prioritize variants based on functional or evolutionary properties such as sequence conservation, genic effects and regulatory element annotations can serve to improve power and ultimately the success of disease studies, for both Mendelian phenotypes (3) as well as common traits and diseases (4).

We previously described ‘Combined Annotation-Dependent Depletion’ or CADD, a score that ranks genetic variants, including single nucleotide variants (SNVs) and short inserts and deletions (InDels), throughout the human genome reference assembly (5). CADD scores are based on diverse genomic features derived from surrounding sequence context, gene model annotations, evolutionary constraint, epigenetic measurements and functional predictions. For any given variant, all of these annotations are integrated into a single CADD score via a machine learning model. For improved interpretability, these are transformed into a PHRED-like (i.e. log_10_-derived, (6)) rank score based on the genome-wide distribution of scores for all ∼9 billion potential SNVs, the set of all three non-reference alleles at each position of the reference assembly.

In contrast to many other approaches, CADD is intentionally *not* trained on the relatively limited number of genomic variants for which pathogenic or benign status is ‘known’. Rather, CADD relies on less biased, much larger training sets. It assumes that variants that have arisen and fixed across humanity since the last human-ape ancestor are mostly benign or neutral since they have persisted despite millions of years of purifying selection; for simplicity, we will refer to these variants as *proxy-neutral.* Such variants are contrasted with a second set of simulated *de novo* variants that are free of selective pressure; while many such variants will also be neutral, an unknown but considerable fraction would likely be deleterious, phenotypically influential mutations if observed in an individual; for simplicity, we will refer to these variants as *proxy-deleterious*. The contrast between the *proxy-neutral* and *proxy-deleterious* variant sets, i.e. the relative paucity of deleterious, phenotypically influential mutations in the *proxy-neutral* set and the resulting differences in their annotation features, is the core characteristic of CADD and motivates its name (‘CADD’).

The key advantages of the CADD framework include systematic and objective labeling of variants for the training set, an ability to accommodate nearly any feature that can be tied to reference assembly coordinates, and the capacity to score both coding and non-coding variants. Each iteration of the CADD model is trained on more than 30 million variants and hundreds of features derived from available annotations. The size of the training set allows integration of many annotations without substantial risk of overfitting.

A limitation of CADD is that the training set label for any given variant (i.e. *proxy-neutral* or *proxy-deleterious*) provides an imperfect approximation of whether the variant is benign versus pathogenic. In particular, an unknown proportion of the *proxy-deleterious* variants are certainly neutral. Consequently, we do not evaluate CADD’s performance (or select its tuning parameters) using a hold-out of the training set. Rather, we rely on curated datasets related to disease or functional effects across both coding and regulatory regions. Examples include the task of discriminating ClinVar pathogenic (7) versus common human genetic variants (8); correlation with experimentally measured functional effects in regulatory elements (9–12); and gene-wide frequencies of somatic mutations in cancer genes (13). In the most recent CADD version, the largest curated datasets were split into two subsets, of which one was used to select tuning parameters for the CADD model, and the other was used to evaluate performance. To summarize, CADD does not rely on manual/subjective variant curation in model training, although manually curated variant sets are used to select tuning parameters and to evaluate the overall performance of CADD.

## CADD FRAMEWORK

**Figure 1.**
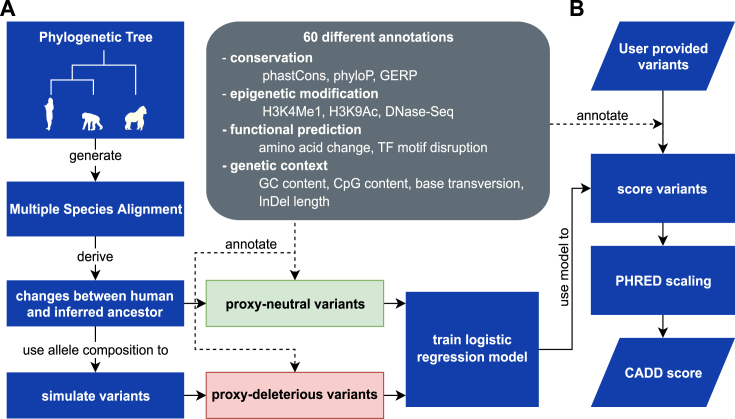
The CADD framework. (**A**) Training a CADD model requires the identification of variants that are fixed or nearly fixed in human populations, but are absent in the inferred genome sequence of the human-ape ancestor (*proxy-neutral* variants). The sequence composition of this variant set is used to draw a matching set of *proxy-deleterious* variants. Using more than 60 diverse annotations, a machine learning model is trained to classify variants as *proxy-neutral* versus *proxy-deleterious*. All potential SNVs of the human reference genome are annotated using the same features, and raw CADD scores are calculated. A PHRED conversion table is derived from the relative ranking of these model scores. (**B**) Users provide variant sets in VCF, and CADD uses the chromosome, position, reference allele and alternative allele columns from these files. Scores are either retrieved from pre-scored files, or else variants are fully annotated and the CADD score is calculated. The PHRED-scaled score is then looked up in the conversion table, and both scores returned to the user. Users may request output files containing variant annotations.

An overview of the CADD method is shown in Figure 1. It consists of a model-fitting phase, followed by a variant-scoring phase. Most CADD users will make use of the model that we have already fit, and hence will interact only with the variant-scoring phase.

In training a CADD model, we first define two variant sets: a *proxy-neutral* set and a *proxy-deleterious* set. The *proxy-neutral* variants have an allele frequency of 95–100% in humans but are absent in the inferred genome sequence of the human-ape ancestor (i.e. human-derived and fixed or nearly fixed; identified from Ensembl EPO (14) whole genome alignments; 15 million SNVs and 1.8 million InDels). The sequence composition of the *proxy-neutral* variants is used to simulate a matching set of *de novo* variants, i.e. the *proxy-deleterious* set.

Using more than 60 different, diverse annotations to derive hundreds of numerical model features, a classification model is trained to separate these two variant sets. Annotations are obtained using Ensembl Variant Effect Predictor (VEP (15)), conservation and selection scores (e.g. PhyloP (16), PhastCons (17), GERP++ (18)), different tracks from the UCSC genome browser (19) as well as flat files of epigenetic information from the ENCODE and NIH RoadMap projects. Annotations span a wide range of data types and are frequently only available for subsets of variants. Examples of annotations include transcript information like distance to exon-intron boundaries, DNase hypersensitivity, transcription factor binding, expression levels in commonly studied cell lines and amino acid substitution scores for protein coding sequences like Grantham (20), SIFT (21) and PolyPhen2 (22). Lists of annotations used in CADD v1.4 are available as Supplementary Tables S1 and S2. For InDels, variant effects are used as predicted from VEP. For all other annotations, the extreme values are selected from the two neighboring positions for insertions and across the bases of the removed range for deletions. After model training, the fitted model is applied to all ∼9 billion potential SNVs of the human reference genome in order to calculate raw CADD scores. A PHRED conversion table is derived from the relative ranking of model scores across all potential SNVs (−10 log_10_ rank/total number of potential substitutions). Details on the different usage of these scores is available in the section ‘Raw versus scaled scores’.

In order to score variants (defined by chromosome, position, reference and alternative allele), users provide variant sets as files in Variant Call Format (VCF), optionally gzip-compressed or look up individual SNVs or SNV coordinate ranges from the pre-scored genome files (see also section on ‘Web access and score availability’). Variant sets can be scored by uploading data to our web server, https://cadd.gs.washington.edu/ or else by using a local CADD installation. In order to upload data to our web server, users must confirm that they are authorized to upload the data, that their upload does not contain any identifiable information, and that they understand that our server does not require user registration and that therefore data is accessible by decrypting URLs. Users, who are unable to confirm this, have the option to score variants offline, using a local CADD installation. Given a variant to be scored from a variant set, the CADD score is either retrieved from an already pre-computed file (e.g. a file of CADD scores for all ∼9 billion potential SNVs) or else obtained by annotating the variant and applying the previously-fitted model. The PHRED-scaled score is looked up in a conversion table and both scores are returned to the user. In addition, the user may request that the output files contain the variant annotations used to create the CADD score.

## RAW VERSUS SCALED SCORES

Two scores are returned to users for each variant. ‘Raw’ scores are the immediate output from the machine learning model. They summarize the extent to which the variant is likely to have derived from the *proxy-neutral* (negative values) or *proxy-deleterious* (positive values) class. Because they have no absolute meaning, they cannot be directly compared across models with distinct annotation combinations, training sets or tuning parameter choices. However, raw scores do have relative meaning, in the sense that higher values indicate that a variant is more likely to have derived from the *proxy-deleterious* than the *proxy-neutral* variant set, and is therefore more likely to have deleterious effects. ‘PHRED-scaled’ scores are normalized to all potential ∼9 billion SNVs, and thereby provide an externally comparable unit for analysis. For example, a scaled score of 10 or greater indicates a raw score in the top 10% of all possible reference genome SNVs, and a score of 20 or greater indicates a raw score in the top 1%, regardless of the details of the annotation set, model parameters, etc.

Raw scores offer superior resolution across the entire spectrum, and preserve relative differences between scores that may otherwise be rounded away in the scaled scores (only six significant digits are retained in the scaled scores). For example, the bottom 90% (∼7.7 billion) of all GRCh37/hg19 reference SNVs (∼8.6 billion) are compressed into scaled CADD units of 0 to 10, while the next 9% (top 10% to top 1%, spanning ∼774 million SNVs) occupy CADD-10 to CADD-20, etc. As a result, many variants that have substantively different raw scores may have very similar, or even the same, scaled scores; and scaled scores accurately resolve differences between variants’ scores only at the extreme top end. Thus, when comparing distributions of scores between groups of variants (e.g. variants seen in cases versus variants seen in controls), raw scores should be used. However, when discovering causal variants or fine-mapping variants within associated loci, scaled scores are advantageous as they allow the user a direct interpretation in terms of the estimated pathogenicity relative to all possible SNVs in the reference genome.

It is tempting to declare a single universal cut-off value for CADD scores, above which a variant is declared ‘pathogenic’ (or ‘functional’ or ‘deleterious’) as opposed to ‘benign’ (or ‘non-functional’ or ‘neutral’) across all datasets. However, we believe that such an approach is flawed for at least two reasons. First, a substantial loss of information would result from binarizing continuous-valued CADD scores. Second, the choice of cut-off would naturally depend on a number of analysis-specific factors, such as the severity of the phenotype, whether the variant is dominant or recessive, and the amount of time and resources available for curation or experimental follow-up of variants. Therefore, we recommend ranking all variants by CADD score, and then further investigating the top-ranked variants to the extent that is meaningful within the given study design or allowed by the available resources for follow-up assessment. However, for an alternative view on this topic, we refer the reader to recent methods that use CADD scores in conjunction with hard cutoffs; see GAVIN (23) and MSC (24). We also note that for better or worse, the binary classification of variants as pathogenic versus benign is still the standard practice (and perhaps the expectation) in the medical genetics field.

## THE IMPACT OF CADD SCORES IN HUMAN GENETICS

The primary use of CADD has been to score variants across the reference genome to identify those that are most likely to be deleterious and potentially pathogenic. Thus, its major application is the prioritization of variants from among thousands to millions of candidates. This includes variants from clinical studies, like *de novo*, dominant and recessive variants discovered in family-based sequencing (e.g. 23,25–28), as well as variants identified in population-based studies (e.g. 29). Since its introduction in 2014, CADD has become one of the most widely used tools to assess human genetic variation, and other tools and scores often use CADD to benchmark their performance; according to Google Scholar CADD has been cited 1984 times (as of 15 September 2018) with about 24 000 unique users of its website over the last year.

Furthermore, CADD has also seen applications in evolutionary studies, ranging from the interpretation of evolutionary changes (30–32) to the theoretical investigation of variant fitness effects in human populations (33).

The release of CADD has also spurred the development of several other genome-wide predictors. For instance, the feature set from CADD has been used to train Deep Neural Networks (e.g. DANN (34)), and CADD’s underlying approach and training set definition methodology has been adapted for other model organisms (35). A similar approach based on ape-lineage-derived variants has been used to score non-synonymous variants (36). CADD has also been used to develop tools for complex variants, like scoring the effect of larger structural variants (e.g. SVScore (37)). Some recently developed predictors are ensemble learners, which combine CADD and other scores (38–41). However, we are not aware of any competing tool for variant-scoring that consistently outperforms CADD in comprehensive testing across diverse use cases in human genetics.

## CADD UPDATES AND SUPPORT OF GRCh38

Since the initial release of CADD in 2014, we have published four score updates. Besides, minor bug fixes and adjustments to the genomic features (Supplementary Table S3), the main change between these releases was the choice of the machine learning algorithm and software library. A major challenge in training a CADD model is the size of the fully annotated training dataset, which comprises hundreds of gigabytes if stored naively. This is difficult to handle in active working memory, and therefore needs to be kept in a sparse matrix representation or handled using other computational techniques. While CADD v1.0 used a linear support vector machine implemented in the LIBOCAS library (42), later models used L_2_-regularized logistic regression implemented in GraphLab Create (43). For the latest release, CADD v1.4, a logistic regression model was fit using a fully open source pipeline based on SciPy (44) and scikit-learn (45). All libraries permit model training in sparse matrix format, with major benefits in terms of run time and memory requirements.

**Figure 2.**
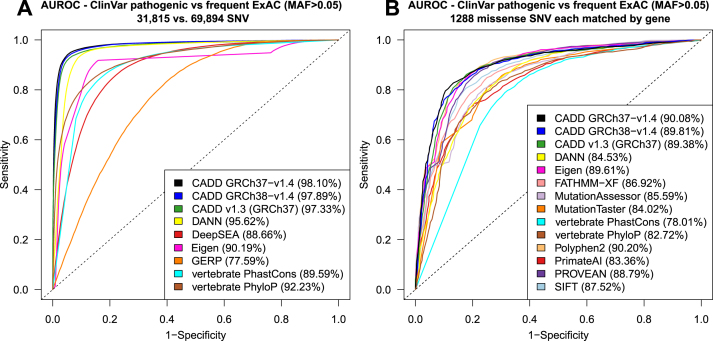
Performance of CADD in comparison to other scores. Different scores are compared by area under the receiver operating characteristic (AUROC) in terms of how well they separate known pathogenic variants (ClinVar pathogenic) from frequent exome variants (ExAC, mean allele frequency >5%, assumed to be neutral): (**A**) All variants of the two sets, and (**B**) missense variants only, with matching genes between the two sets. PolyPhen2 and PROVEAN, two dedicated protein missense variant scores, perform on par with CADD and Eigen, while all other scores have a lower AUROC. The performance of CADD GRCh38-v1.4 is not significantly different from the other CADD releases. The results for more missense scores and non-coding variants are shown in Supplementary Figure S1.

A performance comparison of our latest set of CADD models to other commonly used scores is available in Figure 2. We validate CADD’s ability to separate variants reported to be pathogenic in the NCBI/NIH ClinVar database (7) from common variants (mean allele frequency > 0.05) in the ExAC database (8), including a comparison matching missense variants in the same genes (see Supplementary Materials for more details). We also highlight that CADD score performance extends beyond missense variants and across different variant effect categories, such as those measured by experimental assessments of transcriptional regulatory influence.

**Figure 3.**
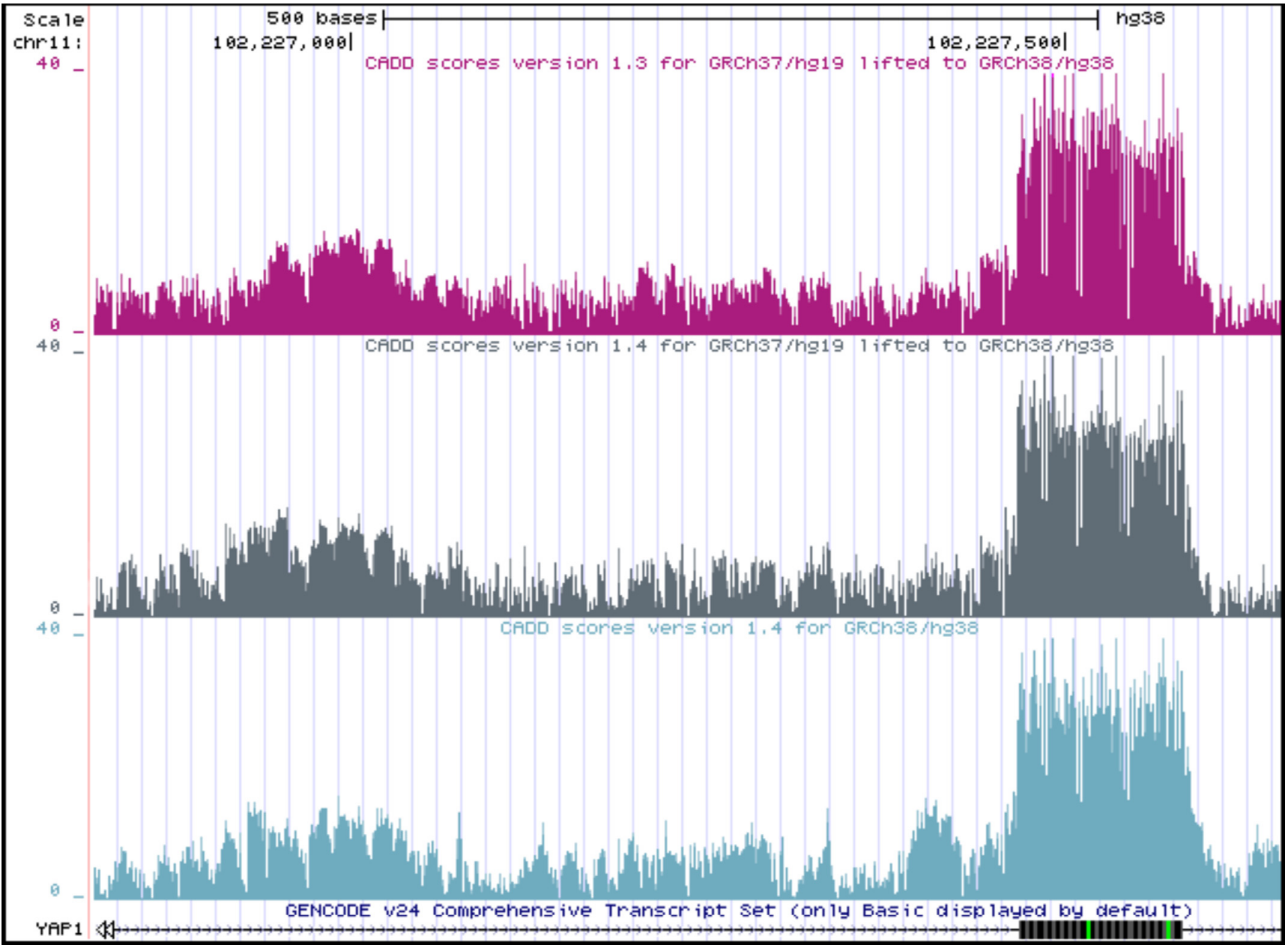
Comparison of CADD v1.3 and v1.4 in the UCSC Genome Browser: CADD GRCh38-v1.4 scores (light blue) in comparison to lifted scores of the models of CADD v1.3 (pink) and v1.4 (gray) originally obtained for the GRCh37 genome build. Each browser track shows the maximum CADD score of the three possible SNVs at each genomic position.

CADD v1.0-v1.3 made use of the human genome build GRCh37. In the latest release, v1.4, we also provide scores for the human genome build GRCh38. Because new annotations primarily support GRCh38, and coordinate liftovers are limited to regions well characterized in both genome builds, the new model is based almost entirely on annotations generated on GRCh38 (see Supplementary Materials). We chose annotations that are identical or similar to those used in the CADD GRCh37-v1.4 model. Although training and parameter optimization were performed independently on GRCh37 and GRCh38 models, for regions well-represented in both genome builds, the fitted models provided very similar variant scores (Figure 3). In total, CADD v1.4 covers 2 937 639 113 bases on GRCh38 compared to 2 858 658 094 bases on GRCh37. When compared through coordinate liftover on a random sample of sites, the two different releases show very similar score distributions with Pearson correlation of 0.79 (Supplementary Figure S2, GRCh37-v1.4 and v1.3 have a Pearson correlation of 0.89).

## WEB ACCESS AND SCORE AVAILABILITY

CADD is available for SNVs as well as InDels shorter than 50 bp located on the 22 human autosomes and chromosome X. We further provide scores for chromosome Y, although not all annotations are available. Due to a lack of available annotations, we currently do not support alternative haplotypes and other contigs. In previous releases, CADD scored variants located on the mitochondrial genome. However, due to differences in inheritance, gene density, transcription machinery and the availability of annotations, we have decided to no longer support scoring of mitochondrial variants.

**Figure 4.**
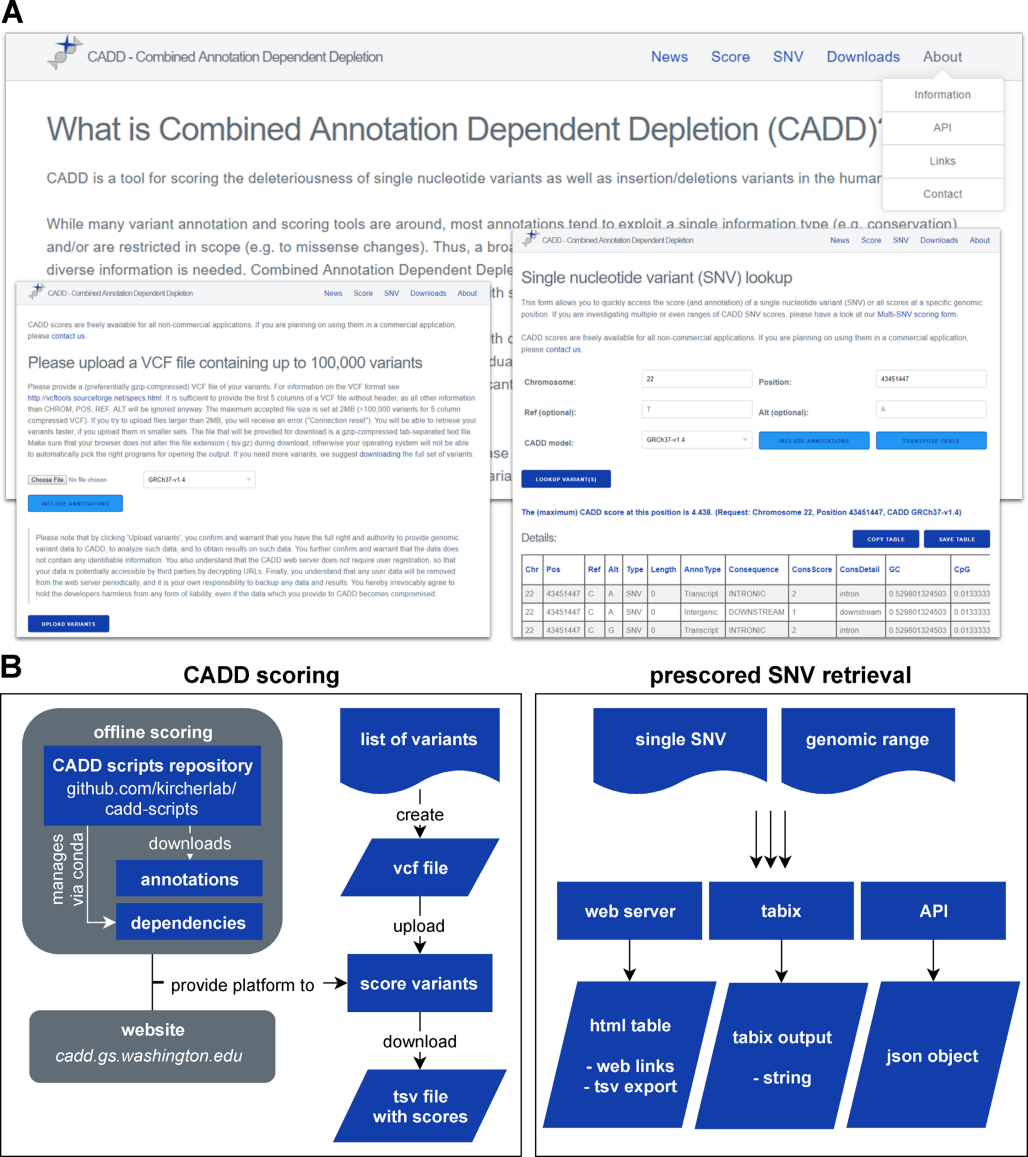
Available CADD services. (**A**) The web server https://www.cadd.gs.washington.edu provides a rich resource for obtaining CADD scores and the underlying annotations on which they are based, as well as scripts, documentation, etc. (**B**) There are several ways to obtain CADD scores. First, CADD scores can be calculated for SNVs and short InDels using offline scripts or our website. Second, pre-scored SNVs and InDels can be obtained from indexed files via the graphical website interface, API or through tabix.

CADD scores, and the associated software, are freely available for all non-commercial applications. They are primarily distributed through our website (https://cadd.gs.washington.edu), but there are a number of different ways to obtain them (Figure 4). With the latest release, we have considerably improved and extended the services provided. As with all prior versions, users can perform scoring of SNVs or short InDels online via upload of a VCF file or can download pre-scored variant sets, including the scores of ∼9 billion potential SNVs created from the human reference sequence. For users only interested in a small number of SNVs, the score lookup process can now be simplified and accelerated by either retrieving pre-scored SNVs via tabix (46), or through a new interface that provides scores and annotations for a single SNV, a genomic coordinate, or ranges thereof. This score lookup also includes further information about variants of interest by linking to external resources like Ensembl (47), NCBI Genome Data Viewer (https://www.ncbi.nlm.nih.gov/genome/gdv/), UCSC Genome Browser or gnomAD.

In order to enable external sources to refer directly to CADD scores, we have enabled direct links to the scores of SNVs, and we now provide an application programming interface (API) to retrieve scores. At last, we also provide bigWig files of the maximum SNV score per genomic position that can be visualized as browser tracks for utilities like the UCSC genome browser (Figure 3) or Integrative Genomics Viewer (IGV), and allow users to screen larger genomic areas quickly.

For users interested in scoring SNV and InDel variants on their own system, we provide software for offline scoring, starting with CADD v1.1. Offline scoring takes a VCF file as input, and allows for retrieval of annotations from pre-scored variant sets, and annotation and scoring of the remaining variants. It returns a gzip-compressed tab-separated text file (tsv.gz) containing all scored variants, with or without annotations. In the latest release, we have simplified the installation process by introducing dependency management through conda (https://conda.io), and providing an installation script that downloads all necessary annotations and, optionally, pre-scored variants. The source code for offline CADD scoring is available on GitHub (https://github.com/kircherlab/cadd-scripts) and open to contribution by others.

In addition, our SNV scores are available through a number of third-party sources, such as dbNSFP (48), as a plug-in for Ensembl VEP, ANNOVAR (49), SeattleSeq (50), ExAC/gnomAD (8) and PopViz (51). We note that at the time of this publication, these third-party sources do not distinguish between CADD for GRCh38 and GRCh37, and may well annotate lifted CADD v1.3 scores to GRCh38 variants.

## FUTURE WORK

In general, integrative annotations like CADD benefit enormously from domain-specific scores such as PolyPhen2 and SIFT, which boost performance in the coding regions of the genome. In the future, we plan to add more domain-specific scores and annotations to advance CADD scores in regions of the genome that are not protein-coding. For example, CADD currently does not include any information about non-coding RNA species besides predicted miRNA binding sites. Of special interest are regulatory variants in promoters, enhancers and near splice sites, as a number of other recent variant classifiers (26,52–55) have shown the potential of predicting regulatory effects from sequence and annotations describing the biological function. Specialized scores derived from functionally testing large numbers of variants via multiplex assays (56,57) may also be integrated into CADD in the near future.

Further improvement of CADD could also come in terms of a more complex, structured model that combines features via linear or non-linear interactions. Currently, CADD includes features obtained by taking the product of VEP-predicted variant consequences with a number of annotations, such as conservation and transcript position. In the future, a more sophisticated and streamlined approach could be applied in order to allow for non-linearity and interactions within CADD. However, this must be performed with care, as the risk of overfitting such complex models is high.

## DISCUSSION

In this manuscript, we presented an overview of recent updates to CADD, as well as the services that we provide in order to make those scores available and maximally useful to the scientific community. In addition to better documentation and a fresh web layout, we substantially expanded the options for how users can access scores by providing website and API lookups, genome browser tracks and an easy-to-install offline scoring script. With the release of CADD v1.4, we support direct (non-lifted) variant interpretation on GRCh38 and show that the available annotations provide a similar level of accuracy to those generated for GRCh37.

A key strength of CADD is that the model is trained on a very large training set that does not suffer from ascertainment bias inherent to curated sets of pathogenic and benign variants such as ClinVar (7) or HGMD (58). CADD shares this strength with only a few other scores, such as Eigen (59), LINSIGHT (60) and CDTS (61). As a general statement, we believe that CADD and tools like it that: (i) integrate many correlated genomic annotations in a principled fashion; (ii) rely on large training datasets to minimize the risk of overfitting; and (iii) avoid curated sets of pathogenic and benign variants during training, represent the best path forward for predicting the relative pathogenicity or functional importance of human genetic variants on a genome-wide basis.

As genomic annotations grow in depth and breadth, CADD and CADD-inspired variant scores will continue to improve and provide utility across a wide range of analytical scenarios. While this is particularly true for studies of Mendelian disease, many complex-trait, comparative genomic, population genetic and functional genomic studies are likely to also benefit from current and future versions of CADD and related frameworks.

## Supplementary Material

Supplementary Data
