# Supplementary material for: CADD: predicting the deleteriousness of variants throughout the human genome
Source: Nucleic Acids Res. 2018 Oct 29;47(Database issue):D886–94. doi: 10.1093/nar/gky1016 (PMC6323892; doi:10.1093/nar/gky1016)
Supplement: Supplementary Data [file gky1016_supplemental_files.pdf]

Supplementary Materials

CADD: Predicting the deleteriousness of variants throughout the human genome

Philipp Rentzsch, Daniela Witten, Gregory M. Cooper, Jay Shendure*, Martin Kircher*

* Correspondence should be addressed to shendure@uw.edu and martin.kircher@bihealth.de

Variant selection and external data sets

Variants for **Figures 2** and **S1** were selected from NCBI/NIH ClinVar and ExAC databases. In the pathogenic set, we collected all ClinVar SNVs with clinical significance "pathogenic" as of July 29th, 2018. In the neutral set, we collected all SNVs from ExAC that pass the variant quality filter and have mean allele frequency of 5-50%. Variants overlapping between the ClinVar and ExAC sets were removed. All variants were coordinate lifted from GRCh37 to GRCh38 and back using CrossMap (1) to obtain a consistent set for both CADD v1.4 models. Overall, we obtained 31,815 pathogenic and 69,894 neutral variants located on all 22 autosomes or chromosome X. From these, all missense variants were selected based on their Ensembl VEP annotation in CADD v1.4. We calculated the number of missense variants per gene for both pathogenic and neutral sets and randomly drew the smaller number per gene from each set for the missense performance test (leaving 1,288 variants in each set). Missing score values were imputed with median values of all other variants in the two sets. Non-coding variants in **Figure S1A** were selected from all variants in the two set that were available in the Eigen, FunSeq2 (2) and LINSIGHT whole-genome files.

Scores for CADD, DeepSEA and FATHMM-XF (3) were obtained through their respective web servers. CDTS, DANN, Eigen (v1.1), FunSeq2 (v2.1.6), GERP, LINSIGHT, PrimateAI and ReMM (v0.3.1) scores were extracted from genome-wide files provided by the respective authors. PhastCons and PhyloP scores for vertebrates (based on 100 species alignment) were downloaded from UCSC genome browser resources. All other scores (i.e. FATHMM (4), LRT(5), MutationAssessor (6), MutationTaster (7), Polyphen2, PROVEAN (8), REVEL, SIFT, SiPhy(9), VEST (10)) were annotated via dbNSFP.

Annotations in CADD GRCh38

Gene annotations: same processing as for GRCh37 via VEP

Grantham: not specific to genome build

phastCons, phyloP, GERP++: novel score generated based on multiz100way alignment of hg38 from UCSC genome browser (excluding the human genome sequence in calculation)

bstatistic (11): liftover from hg18

mirSVR (12): liftover from hg19

targetScan (13,14): liftover from hg19

chromHMM (15): uses chromHMM 25 state model, ENCODE/Roadmap cell types only

Encode expression, nucleosome position, histone modification, open chromatin: Encode reference epigenome data from up to 14 cell types for totalRNAseq, DNase-seq and Chip-Seq of 10 different histone modifications, for each we include one feature with sum across cell types and one feature with maximum across cell types; all signals were log-transformed

Segway (16): replaced with Ensembl regulatory build (which is based on a Segway model)

tOverlapMotifs: liftover from hg19

TFBS: replaced by data from ReMap2 (17), we are counting for every genomic position the number of different TF and the number of TF-cell-type hits

mutationDensity, nearestMutation: same processing based on BRAVO/TOPMed freeze 5

dbscSNV (18): liftover from hg19

Known variants in CADD

In interacting with our users, we noted confusion about whether and how CADD integrates information about known variants into its prediction. CADD v1.0 to v1.3 never used information of variant presence, observed population frequencies or patient phenotype derived effect predictions. We only reported variant presence as well as Exome Sequencing Project (ESP) and 1000 Genomes allele frequencies in our annotation files for the convenience of evaluating CADD's performance.

Since recent studies have highlighted the value of variant density information (21), we have added features based on gnomAD/BRAVO in CADD version 1.4. One feature is the distance between the next single nucleotide variants up and downstream (ignoring variants at the site itself). For the second feature set, we count the number of frequent (MAF > 0.05), rare and single occurrence single nucleotide variants in a window of 100, 1000 and 10000 bases around the position of interest. Considering the use of linear models, it is not possible to infer variable positions from the combination of these accumulated counts and the distance feature. Hence, variant frequencies can be used as a potentially orthogonal annotation by CADD users.

Application Program Interface

In addition to the retrieving CADD scores via our graphical web user interface and offline scoring, it is possible to request SNV scores per variant or for short ranges via an Application Program Interface (API).

All API requests consist of a CADD version and the genome coordinate. The available CADD versions are `v1.0` to `v1.3` and the two v1.4 releases `GRCh37-v1.4` and `GRCh38-v1.4`. If you require annotations, you can add `_inclAnno` to the version string.

Single position access: The request path for SNV access is `https://cadd.gs.washington.edu/api/v1.0/<CADD-version>/<chrom>:<pos>` which returns a JSON list of the three SNVs at that position. Users can request a single SNV with reference and alternate base given via `https://cadd.gs.washington.edu/api/v1.0/<CADD-version>/<chrom>:<pos>_<ref>_<alt>`. This returns just a single SNV object in a list. In cases where ref or alt are not available, an empty list is returned.

Range access: Range access is similar to the SNV range access on the website with the same limitation to 100 contiguous bases. It can be accessed via `https://cadd.gs.washington.edu/api/v1.0/<chrom>:<start>-<end>`. In contrast to the single position access, this returns a list of lists where the first item contains the field names.

Currently, there is no support for retrieving CADD InDel scores through the API.

Figure S1

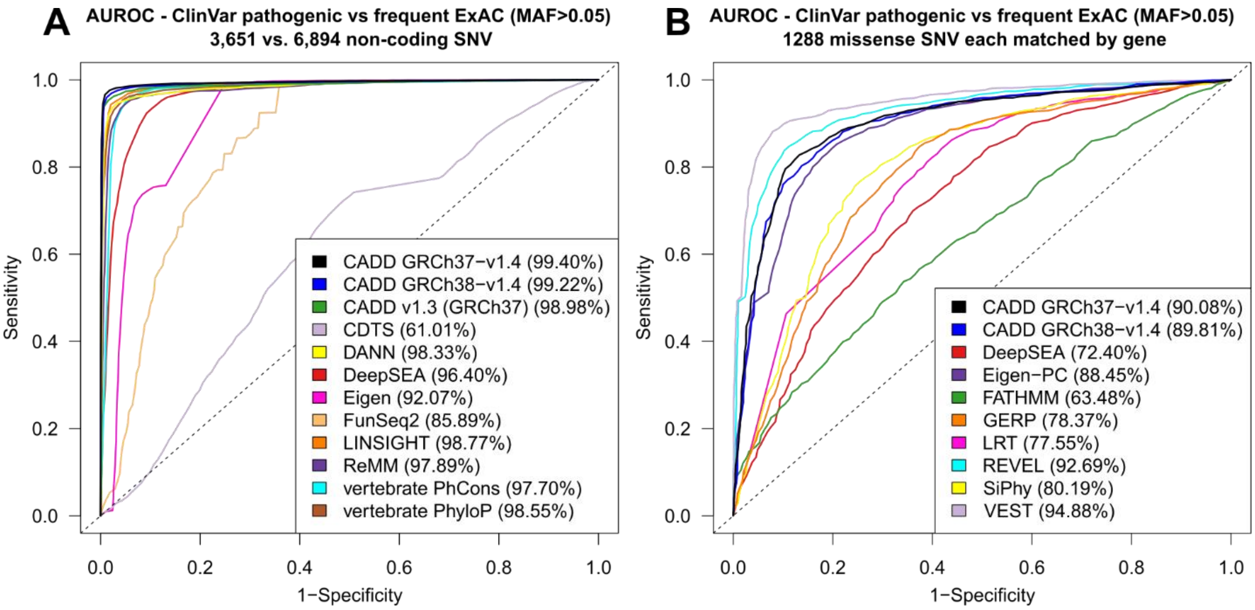

Non-coding variants and more missense scores: AUROC performances of different scores on (A) non-coding variants and (B) the same missense variants from **Figure 2B**. Scores from VEST and REVEL are not included in the main comparison because they were trained on variants from these two test sets.

Figure S2:

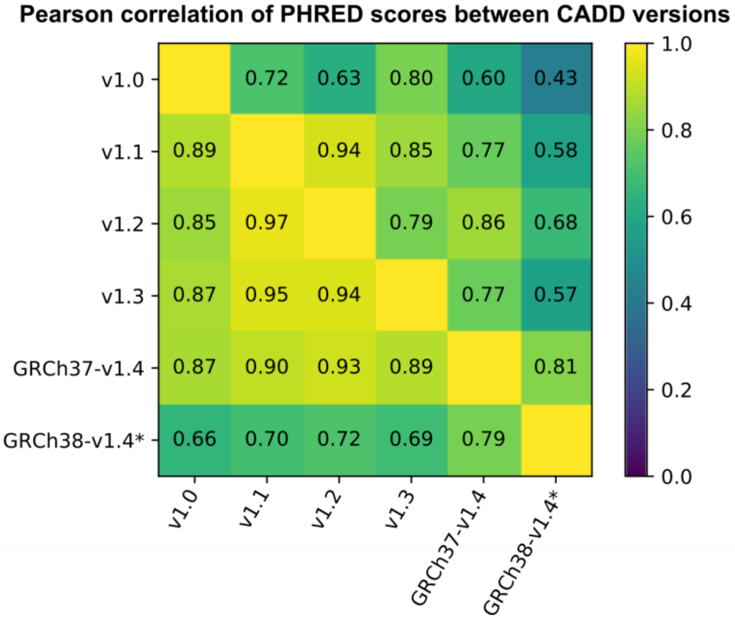

Correlation between CADD versions: The plot shows Pearson correlation coefficients of PHRED-scaled scores between the latest six CADD models. GRCh38 scores were lifted to GRCh37 for comparability. Since annotations are not perfectly correlated between genome builds, the GRCh38-v1.4 model is the least correlated to the other models. The figure is based on 100,000 randomly selected SNV throughout the genome (below the diagonal) and only those with PHRED-scaled scores greater than 10 in CADD GRCh37-v1.4 (n=9,923, above the diagonal).

Table S1: Annotation columns in CADD GRCh37-v1.4

	Name	Type	Description
1	(Chrom)	String	Chromosome
2	(Pos)	integer	Position (1-based)
3	Ref	factor	Reference allele (default: N)
4	Alt	factor	Observed allele (default: N)
5	Type	factor	Event type (SNV, DEL, INS)
6	Length	integer	Number of inserted/deleted bases
7	(Annotype)	factor	CodingTranscript, Intergenic, MotifFeature, NonCodingTranscript, RegulatoryFeature, Transcript
8	Consequence	factor	VEP consequence, priority selected by potential impact (default: UNKNOWN)
9	(ConsScore)	integer	Custom deleterious score assigned to Consequence
10	(ConsDetail)	string	Trimmed VEP consequence prior to simplification
11	GC	float	Percent GC in a window of +/- 75bp (default: 0.42)
12	CpG	float	Percent CpG in a window of +/- 75bp (default: 0.02)
13	MotifECount	integer	Total number of overlapping motifs (default: 0)
14	(MotifEName)	string	Name of sequence motif the position overlaps
15	MotifEHIPos	bool	Is the position considered highly informative for an overlapping motif by VEP (default: 0)
16	MotifEScoreChng	float	VEP score change for the overlapping motif site (default: 0)
17	oAA	factor	Reference amino acid (default: unknown)
18	nAA	factor	Amino acid of observed variant (default: unknown)
19	(GeneID)	string	ENSEMBL GeneID
20	(FeatureID)	string	ENSEMBL feature ID (Transcript ID or regulatory feature ID)
21	(GeneName)	string	GeneName provided in ENSEMBL annotation
22	(CCDS)	string	Consensus Coding Sequence ID
23	(Intron)	string	Intron number/Total number of exons
24	(Exon)	string	Exon number/Total number of exons
25	cDNApos	float	Base position from transcription start (default: 0*)
26	relcDNApos	float	Relative position in transcript (default: 0)
27	CDSpos	float	Base position from coding start (default: 0*)
28	relCDSpos	float	Relative position in coding sequence (default: 0)
29	protPos	float	Amino acid position from coding start (default: 0*)
30	relprotPos	float	Relative position in protein codon (default: 0)
31	Domain	factor	Domain annotation inferred from VEP annotation (ncoils, sigp, lcompl, hmmpanther, ndomain = "other named domain") (default: UD)
32	Dst2Splice	float	Distance to splice site in 20bp; positive: exonic, negative: intronic (default: 0)
33	Dst2SplType	factor	Closest splice site is ACCEPTOR or DONOR (default: unknown)
34	MinDistTSS	float	Distance to closest Transcribed Sequence Start (TSS) (default: 5.5)
35	MinDistTSE	float	Distance to closest Transcribed Sequence End (TSE) (default: 5.5)
36	SIFTcat	factor	SIFT category of change (default: UD)
37	SIFTval	float	SIFT score (default: 0*)
38	PolyPhenCat	factor	PolyPhen2 category of change (default: UD)
39	PolyPhenVal	float	PolyPhen2 score (default: 0*)

40	priPhCons	float	Primate PhastCons conservation score (excl. human) (default: 0.115)
41	mamPhCons	float	Mammalian PhastCons conservation score (excl. human) (default: 0.079)
42	verPhCons	float	Vertebrate PhastCons conservation score (excl. human) (default: 0.094)
43	priPhyloP	float	Primate PhyloP score (excl. human) (default: -0.033)
44	mamPhyloP	float	Mammalian PhyloP score (excl. human) (default: -0.038)
45	verPhyloP	float	Vertebrate PhyloP score (excl. human) (default: 0.017)
46	bStatistic	integer	Background selection score (default: 800)
47	targetScan	integer	targetscan (default: 0*)
48	mirSVR-Score	float	mirSVR-Score (default: 0*)
49	mirSVR-E	float	mirSVR-E (default: 0)
50	mirSVR-Aln	integer	mirSVR-Aln (default: 0)
51	cHmmTssA	float	Proportion of 127 cell types in cHmmTssA state (default: 0.0667*)
52	cHmmTssAFlnk	float	Proportion of 127 cell types in cHmmTssAFlnk state (default: 0.0667)
53	cHmmTxFlnk	float	Proportion of 127 cell types in cHmmTxFlnk state (default: 0.0667)
54	cHmmTx	float	Proportion of 127 cell types in cHmmTx state (default: 0.0667)
55	cHmmTxWk	float	Proportion of 127 cell types in cHmmTxWk state (default: 0.0667)
56	cHmmEnhG	float	Proportion of 127 cell types in cHmmEnhG state (default: 0.0667)
57	cHmmEnh	float	Proportion of 127 cell types in cHmmEnh state (default: 0.0667)
58	cHmmZnfRpts	float	Proportion of 127 cell types in cHmmZnfRpts state (default: 0.0667)
59	cHmmHet	float	Proportion of 127 cell types in cHmmHet state (default: 0.0667)
60	cHmmTssBiv	float	Proportion of 127 cell types in cHmmTssBiv state (default: 0.0667)
61	cHmmBivFlnk	float	Proportion of 127 cell types in cHmmBivFlnk state (default: 0.0667)
62	cHmmEnhBiv	float	Proportion of 127 cell types in cHmmEnhBiv state (default: 0.0667)
63	cHmmReprPC	float	Proportion of 127 cell types in cHmmReprPC state (default: 0.0667)
64	cHmmReprPCWk	float	Proportion of 127 cell types in cHmmReprPCWk state (default: 0.0667)
65	cHmmQuies	float	Proportion of 127 cell types in cHmmQuies state (default: 0.0667)
66	GerpRS	float	Gerp element score (default: 0)
67	GerpRSpval	float	Gerp element p-Value (default: 0)
68	GerpN	float	Neutral evolution score defined by GERP++ (default: 1.91)
69	GerpS	float	Rejected Substitution score defined by GERP++ (default: -0.2)
70	TFBS	float	Number of different overlapping ChIP transcription factor binding sites (default: 0)
71	TFBSPeaks	float	Number of overlapping ChIP transcription factor binding site peaks summed over different cell types/tissue (default: 0)
72	TFBSPeaksMax	float	Maximum value of overlapping ChIP transcription factor binding site peaks across cell types/tissue (default: 0)
73	tOverlapMotifs	float	Number of overlapping predicted TF motifs (default: 0)
74	motifDist	float	Reference minus alternate allele difference in nucleotide frequency within an predicted overlapping motif (default: 0)
75	Segway	factor	Result of genomic segmentation algorithm (default: unknown)
76	EncH3K27Ac	float	Maximum ENCODE H3K27 acetylation level (default: 0)
77	EncH3K4Me1	float	Maximum ENCODE H3K4 methylation level (default: 0)
78	EncH3K4Me3	float	Maximum ENCODE H3K4 trimethylation level (default: 0)
79	EncExp	float	Maximum ENCODE expression value (default: 0)
80	EncNucleo	float	Maximum of ENCODE Nucleosome position track score (default: 0)

81	EncOCC	integer	ENCODE open chromatin code (default: 5)
82	EncOCCombPVal	float	ENCODE combined p-Value (PHRED-scale) of Faire, Dnase, polII, CTCF, Myc evidence for open chromatin (default: 0)
83	EncOCDNasePVal	float	p-Value (PHRED-scale) of Dnase evidence for open chromatin (default: 0)
84	EncOCFairePVal	float	p-Value (PHRED-scale) of Faire evidence for open chromatin (default: 0)
85	EncOCpolIIPVal	float	p-Value (PHRED-scale) of polII evidence for open chromatin (default: 0)
86	EncOCctcfPVal	float	p-Value (PHRED-scale) of CTCF evidence for open chromatin (default: 0)
87	EncOCmycPVal	float	p-Value (PHRED-scale) of Myc evidence for open chromatin (default: 0)
88	EncOCDNaseSig	float	Peak signal for Dnase evidence of open chromatin (default: 0)
89	EncOCFaireSig	float	Peak signal for Faire evidence of open chromatin (default: 0)
90	EncOCpolIISig	float	Peak signal for polII evidence of open chromatin (default: 0)
91	EncOCctcfSig	float	Peak signal for CTCF evidence of open chromatin (default: 0)
92	EncOCmycSig	float	Peak signal for Myc evidence of open chromatin (default: 0)
93	Grantham	float	Grantham score: oAA,nAA (default: 0*)
94	Dist2Mutation	float	Distance between the closest gnomAD SNV up and downstream (position itself excluded) (default: 0*)
95	Freq100bp	integer	Number of frequent (MAF > 0.05) gnomAD SNV in 100 bp window nearby (default: 0)
96	Rare100bp	integer	Number of rare (MAF < 0.05) gnomAD SNV in 100 bp window nearby (default: 0)
97	Sngl100bp	integer	Number of single occurrence gnomAD SNV in 100 bp window nearby (default: 0)
98	Freq1000bp	integer	Number of frequent (MAF > 0.05) gnomAD SNV in 1000 bp window nearby (default: 0)
99	Rare1000bp	integer	Number of rare (MAF < 0.05) gnomAD SNV in 1000 bp window nearby (default: 0)
100	Sngl1000bp	integer	Number of single occurrence gnomAD SNV in 1000 bp window nearby (default: 0)
101	Freq10000bp	integer	Number of frequent (MAF > 0.05) gnomAD SNV in 10000 bp window nearby (default: 0)
102	Rare10000bp	integer	Number of rare (MAF < 0.05) gnomAD SNV in 10000 bp window nearby (default: 0)
103	Sngl10000bp	integer	Number of single occurrence gnomAD SNV in 10000 bp window nearby (default: 0)
104	dbscSNV-ada_score	float	Adaboost classifier score from dbscSNV (default: 0*)
105	dbscSNV-rf_score	float	Random forest classifier score from dbscSNV (default: 0*)
106	RawScore	float	Raw score from the model
107	PHRED	float	CADD PHRED Score

* A Boolean indicator variable was created in order to handle undefined values. Note that often indicators represent more than one annotation. They are created for only (the first) one if the covered genomic regions are identical.

Table S2: Annotations in CADD GRCh38-v1.4

	Name	Type	Description
1	(Chrom)	string	Chromosome
2	(Pos)	integer	Position (1-based)
3	Ref	factor	Reference allele (default: N)
4	Alt	factor	Observed allele (default: N)
5	Type	factor	Event type (SNV, DEL, INS)
6	Length	integer	Number of inserted/deleted bases
7	(AnnoType)	factor	CodingTranscript, Intergenic, MotifFeature, NonCodingTranscript, RegulatoryFeature, Transcript
8	Consequence	factor	VEP consequence, priority selected by potential impact (default: UNKNOWN)
9	(ConsScore)	integer	Custom deleterious score assigned to Consequence
10	(ConsDetail)	string	Trimmed VEP consequence prior to simplification
11	GC	float	Percent GC in a window of +/- 75bp (default: 0.42)
12	CpG	float	Percent CpG in a window of +/- 75bp (default: 0.02)
13	motifECount	integer	Total number of overlapping motifs (default: 0)
14	(motifEName)	string	Name of sequence motif the position overlaps
15	motifEHIPos	bool	Is the position considered highly informative for an overlapping motif by VEP (default: 0)
16	motifEScoreChng	float	VEP score change for the overlapping motif site (default: 0)
17	oAA	factor	Reference amino acid (default: unknown)
18	nAA	factor	Amino acid of observed variant (default: unknown)
19	(GeneID)	string	ENSEMBL GeneID
20	(FeatureID)	string	ENSEMBL feature ID (Transcript ID or regulatory feature ID)
21	(GeneName)	string	GeneName provided in ENSEMBL annotation
22	(CCDS)	string	Consensus Coding Sequence ID
23	(Intron)	string	Intron number/Total number of exons
24	(Exon)	string	Exon number/Total number of exons
25	cDNApos	float	Base position from transcription start (default: 0*)
26	relcDNApos	float	Relative position in transcript (default: 0)
27	CDSpos	float	Base position from coding start (default: 0*)
28	relCDSpos	float	Relative position in coding sequence (default: 0)
29	protPos	float	Amino acid position from coding start (default: 0*)
30	relProtPos	float	Relative position in protein codon (default: 0)
31	Domain	factor	Domain annotation inferred from VEP annotation (ncoils, sigp, lcompl, hmmpanther, ndomain = "other named domain") (default: UD)
32	Dst2Splice	float	Distance to splice site in 20bp; positive: exonic, negative: intronic (default: 0)
33	Dst2SplType	factor	Closest splice site is ACCEPTOR or DONOR (default: unknown)
34	minDistTSS	float	Distance to closest Transcribed Sequence Start (TSS) (default: 5.5)
35	minDistTSE	float	Distance to closest Transcribed Sequence End (TSE) (default: 5.5)
36	SIFTcat	factor	SIFT category of change (default: UD)
37	SIFTval	float	SIFT score (default: 0*)

38	PolyPhenCat	factor	PolyPhen2 category of change (default: UD)
39	PolyPhenVal	float	PolyPhen2 score (default: 0*)
40	priPhCons	float	Primate PhastCons conservation score (excl. human) (default: 0.0)
41	mamPhCons	float	Mammalian PhastCons conservation score (excl. human) (default: 0.0)
42	verPhCons	float	Vertebrate PhastCons conservation score (excl. human) (default: 0.0)
43	priPhyloP	float	Primate PhyloP score (excl. human) (default: -0.029)
44	mamPhyloP	float	Mammalian PhyloP score (excl. human) (default: -0.005)
45	verPhyloP	float	Vertebrate PhyloP score (excl. human) (default: 0.042)
46	bStatistic	integer	Background selection score (default: 800)
47	targetScan	integer	targetscan (default: 0*)
48	mirSVR-Score	float	mirSVR-Score (default: 0*)
49	mirSVR-E	float	mirSVR-E (default: 0)
50	mirSVR-Aln	integer	mirSVR-Aln (default: 0)
51	cHmm_E1	float	Number of 48 cell types in chromHMM state E1_poised (default: 1.92*)
52	cHmm_E2	float	Number of 48 cell types in chromHMM state E2_repressed (default: 1.92)
53	cHmm_E3	float	Number of 48 cell types in chromHMM state E3_dead (default: 1.92)
54	cHmm_E4	float	Number of 48 cell types in chromHMM state E4_dead (default: 1.92)
55	cHmm_E5	float	Number of 48 cell types in chromHMM state E5_repressed (default: 1.92)
56	cHmm_E6	float	Number of 48 cell types in chromHMM state E6_repressed (default: 1.92)
57	cHmm_E7	float	Number of 48 cell types in chromHMM state E7_weak (default: 1.92)
58	cHmm_E8	float	Number of 48 cell types in chromHMM state E8_gene (default: 1.92)
59	cHmm_E9	float	Number of 48 cell types in chromHMM state E9_gene (default: 1.92)
60	cHmm_E10	float	Number of 48 cell types in chromHMM state E10_gene (default: 1.92)
61	cHmm_E11	float	Number of 48 cell types in chromHMM state E11_gene (default: 1.92)
62	cHmm_E12	float	Number of 48 cell types in chromHMM state E12_distal (default: 1.92)
63	cHmm_E13	float	Number of 48 cell types in chromHMM state E13_distal (default: 1.92)
64	cHmm_E14	float	Number of 48 cell types in chromHMM state E14_distal (default: 1.92)
65	cHmm_E15	float	Number of 48 cell types in chromHMM state E15_weak (default: 1.92)
66	cHmm_E16	float	Number of 48 cell types in chromHMM state E16_tss (default: 1.92)
67	cHmm_E17	float	Number of 48 cell types in chromHMM state E17_proximal (default: 1.92)
68	cHmm_E18	float	Number of 48 cell types in chromHMM state E18_proximal (default: 1.92)
69	cHmm_E19	float	Number of 48 cell types in chromHMM state E19_tss (default: 1.92)
70	cHmm_E20	float	Number of 48 cell types in chromHMM state E20_poised (default: 1.92)
71	cHmm_E21	float	Number of 48 cell types in chromHMM state E21_dead (default: 1.92)
72	cHmm_E22	float	Number of 48 cell types in chromHMM state E22_repressed (default: 1.92)
73	cHmm_E23	float	Number of 48 cell types in chromHMM state E23_weak (default: 1.92)
74	cHmm_E24	float	Number of 48 cell types in chromHMM state E24_distal (default: 1.92)
75	cHmm_E25	float	Number of 48 cell types in chromHMM state E25_distal (default: 1.92)
76	GerpRS	float	Gerp element score (default: 0)
77	GerpRSpval	float	Gerp element p-Value (default: 0)
78	GerpN	float	Neutral evolution score defined by GERP++ (default: 3.0)
79	GerpS	float	Rejected Substitution score defined by GERP++ (default: -0.2)
80	tOverlapMotifs	float	Number of overlapping predicted TF motifs

81	motifDist	float	Reference minus alternate allele difference in nucleotide frequency within an predicted overlapping motif (default: 0)
82	EncodeH3K4me1-sum	float	Sum of Encode H3K4me1 levels (from 13 cell lines) (default: 0.76)
83	EncodeH3K4me1-max	float	Maximum Encode H3K4me1 level (from 13 cell lines) (default: 0.37)
84	EncodeH3K4me2-sum	float	Sum of Encode H3K4me2 levels (from 14 cell lines) (default: 0.73)
85	EncodeH3K4me2-max	float	Maximum Encode H3K4me2 level (from 14 cell lines) (default: 0.37)
86	EncodeH3K4me3-sum	float	Sum of Encode H3K4me3 levels (from 14 cell lines) (default: 0.81)
87	EncodeH3K4me3-max	float	Maximum Encode H3K4me3 level (from 14 cell lines) (default: 0.38)
88	EncodeH3K9ac-sum	float	Sum of Encode H3K9ac levels (from 13 cell lines) (default: 0.82)
89	EncodeH3K9ac-max	float	Maximum Encode H3K9ac level (from 13 cell lines) (default: 0.41)
90	EncodeH3K9me3-sum	float	Sum of Encode H3K9me3 levels (from 14 cell lines) (default: 0.81)
91	EncodeH3K9me3-max	float	Maximum Encode H3K9me3 level (from 14 cell lines) (default: 0.38)
92	EncodeH3K27ac-sum	float	Sum of Encode H3K27ac levels (from 14 cell lines) (default: 0.74)
93	EncodeH3K27ac-max	float	Maximum Encode H3K27ac level (from 14 cell lines) (default: 0.36)
94	EncodeH3K27me3-sum	float	Sum of Encode H3K27me3 levels (from 14 cell lines) (default: 0.93)
95	EncodeH3K27me3-max	float	Maximum Encode H3K27me3 level (from 14 cell lines) (default: 0.47)
96	EncodeH3K36me3-sum	float	Sum of Encode H3K36me3 levels (from 10 cell lines) (default: 0.71)
97	EncodeH3K36me3-max	float	Maximum Encode H3K36me3 level (from 10 cell lines) (default: 0.39)
98	EncodeH3K79me2-sum	float	Sum of Encode H3K79me2 levels (from 13 cell lines) (default: 0.64)
99	EncodeH3K79me2-max	float	Maximum Encode H3K79me2 level (from 13 cell lines) (default: 0.34)
100	EncodeH4K20me1-sum	float	Sum of Encode H4K20me1 levels (from 11 cell lines) (default: 0.88)
101	EncodeH4K20me1-max	float	Maximum Encode H4K20me1 level (from 11 cell lines) (default: 0.47)
102	EncodeH2AFZ-sum	float	Sum of Encode H2AFZ levels (from 13 cell lines) (default: 0.9)
103	EncodeH2AFZ-max	float	Maximum Encode H2AFZ level (from 13 cell lines) (default: 0.42)
104	EncodeDNase-sum	float	Sum of Encode DNase-seq levels (from 12 cell lines) (default: 0.0)
105	EncodeDNase-max	float	Maximum Encode DNase-seq level (from 12 cell lines) (default: 0.0)
106	EncodetotalRNA-sum	float	Sum of Encode totalRNA-seq levels (from 10 cell lines always minus and plus strand) (default: 0.0)
107	EncodetotalRNA-max	float	Maximum Encode totalRNA-seq level (from 10 cell lines, minus and plus strand separately) (default: 0.0)
108	Grantham	float	Grantham score: oAA,nAA (default: 0*)
109	Dist2Mutation	float	Distance between the closest BRAVO SNV up and downstream (position itself excluded) (default: 0*)
110	Freq100bp	integer	Number of frequent (MAF > 0.05) BRAVO SNV in 100 bp window nearby (default: 0)
111	Rare100bp	integer	Number of rare (MAF < 0.05) BRAVO SNV in 100 bp window nearby (default: 0)
112	Sngl100bp	integer	Number of single occurrence BRAVO SNV in 100 bp window nearby (default: 0)
113	Freq1000bp	integer	Number of frequent (MAF > 0.05) BRAVO SNV in 1000 bp window nearby (default: 0)
114	Rare1000bp	integer	Number of rare (MAF < 0.05) BRAVO SNV in 1000 bp window nearby (default: 0)
115	Sngl1000bp	integer	Number of single occurrence BRAVO SNV in 1000 bp window nearby (default: 0)
116	Freq10000bp	integer	Number of frequent (MAF > 0.05) BRAVO SNV in 10000 bp window nearby (default: 0)

117	Rare10000bp	integer	Number of rare (MAF < 0.05) BRAVO SNV in 10000 bp window nearby (default: 0)
118	Sngl10000bp	integer	Number of single occurrence BRAVO SNV in 10000 bp window nearby (default: 0)
119	EnsembleRegulatory-Feature	factor	Matches in the Ensemble Regulatory Built (similar to annotype) (default: NA)
120	dbscSNV-ada_score	float	Adaboost classifier score from dbscSNV (default: 0*)
121	dbscSNV-rf_score	float	Random forest classifier score from dbscSNV (default: 0*)
122	RemapOverlapTF	integer	Remap number of different transcription factors binding (default: -0.5)
123	RemapOverlapCL	integer	Remap number of different transcription factor - cell line combinations binding (default: -0.5)
124	RawScore	float	Raw score from the model
125	PHRED	float	CADD PHRED Score

* A Boolean indicator variable was created in order to handle undefined values. Note that often indicators represent more than one annotation. They are created for only (the first) one if the covered genomic regions are identical.

Table S3: Changes in the annotations

Annotation	Introduced	Citation/Comment
ChromHMM	v1.1	(15)
DNA shape factor	v1.1	(19), Led to overfitting and has unclear definition for InDels, therefore removed in v1.4
miRNA binding site prediction (mirSVR & targetScan)	v1.1	(12) & (13,14)
Mutation Index	v1.1	(20), Removed in v1.4 as it uses human reference sequence (and derived alleles) in its calculation.
VEP domain annotation	v1.1	
Mutation density (gnomAD)	v1.4	Based on BRAVO in GRCh38
Splice prediction (dbscSNV)	v1.4	(18)

References

1. Zhao,H., Sun,Z., Wang,J., Huang,H., Kocher,J. and Wang,L. (2014) CrossMap: a versatile tool for coordinate conversion between genome assemblies. *Bioinformatics*, **30**, 1006–1007.

2. Fu,Y., Liu,Z., Lou,S., Bedford,J., Mu,X.J., Yip,K.Y., Khurana,E. and Gerstein,M. (2014) FunSeq2: a framework for prioritizing noncoding regulatory variants in cancer. *Genome Biol.*, **15**, 480.

3. Rogers,M.F., Shihab,H.A., Mort,M., Cooper,D.N., Gaunt,T.R. and Campbell,C. FATHMM-XF: accurate prediction of pathogenic point mutations via extended features. *Bioinformatics*, 10.1093/bioinformatics/btx536.

4. Shihab,H.A., Gough,J., Mort,M., Cooper,D.N., Day,I.N.M. and Gaunt,T.R. (2014) Ranking non-synonymous single nucleotide polymorphisms based on disease concepts. *Hum. Genomics*, **8**, 11.

5. Chun,S. and Fay,J.C. (2009) Identification of deleterious mutations within three human genomes. *Genome Res.*, **19**, 1553–1561.

6. Reva,B., Antipin,Y. and Sander,C. (2011) Predicting the functional impact of protein mutations: application to cancer genomics. *Nucleic Acids Res*, **39**, e118–e118.

7. Schwarz,J.M., Cooper,D.N., Schuelke,M. and Seelow,D. (2014) MutationTaster2: mutation prediction for the deep-sequencing age. *Nat Meth*, **11**, 361–362.

8. Choi,Y., Sims,G.E., Murphy,S., Miller,J.R. and Chan,A.P. (2012) Predicting the Functional Effect of Amino Acid Substitutions and Indels. *PLOS ONE*, **7**, e46688.

9. Garber,M., Guttman,M., Clamp,M., Zody,M.C., Friedman,N. and Xie,X. (2009) Identifying novel constrained elements by exploiting biased substitution patterns. *Bioinformatics*, **25**, i54–i62.

10. Douville,C., Masica,D.L., Stenson,P.D., Cooper,D.N., Gygax,D.M., Kim,R., Ryan,M. and Karchin,R. (2016) Assessing the Pathogenicity of Insertion and Deletion Variants with the Variant Effect Scoring Tool (VEST-Indel). *Human Mutation*, **37**, 28–35.

11. McVicker,G., Gordon,D., Davis,C. and Green,P. (2009) Widespread Genomic Signatures of Natural Selection in Hominid Evolution. *PLOS Genetics*, **5**, e1000471.

12. Betel,D., Koppal,A., Agius,P., Sander,C. and Leslie,C. (2010) Comprehensive modeling of microRNA targets predicts functional non-conserved and non-canonical sites. *Genome Biology*, **11**, R90.

13. Lewis,B.P., Burge,C.B. and Bartel,D.P. (2005) Conserved seed pairing, often flanked by adenosines, indicates that thousands of human genes are microRNA targets. *Cell*, **120**, 15–20.

14. Agarwal,V., Bell,G.W., Nam,J. and Bartel,D.P. (2015) Predicting effective microRNA target sites in mammalian mRNAs. *eLife*, 10.7554/eLife.05005.

15. Ernst,J. and Kellis,M. (2017) Chromatin-state discovery and genome annotation with ChromHMM. *Nature Protocols*, **12**, nprot.2017.124.

16. Hoffman,M.M., Buske,O.J., Wang,J., Weng,Z., Bilmes,J.A. and Noble,W.S. (2012) Unsupervised pattern discovery in human chromatin structure through genomic segmentation. *Nat. Methods*, **9**, 473–476.

17. Chèneby,J., Gheorghe,M., Artufel,M., Mathelier,A. and Ballester,B. (2017) ReMap 2018: an updated atlas of regulatory regions from an integrative analysis of DNA-binding ChIP-seq

experiments. *Nucleic Acids Research*, 10.1093/nar/gkx1092.

18. Jian,X., Boerwinkle,E. and Liu,X. (2014) In silico prediction of splice-altering single nucleotide variants in the human genome. *Nucleic Acids Res*, **42**, 13534–13544.

19. Zhou,T., Yang,L., Lu,Y., Dror,I., Dantas Machado,A.C., Ghane,T., Di Felice,R. and Rohs,R. (2013) DNAshape: a method for the high-throughput prediction of DNA structural features on a genomic scale. *Nucleic Acids Res.*, **41**, W56-62.

20. Michaelson,J.J., Shi,Y., Gujral,M., Zheng,H., Malhotra,D., Jin,X., Jian,M., Liu,G., Greer,D., Bhandari,A., *et al.* (2012) Whole-Genome Sequencing in Autism Identifies Hot Spots for De Novo Germline Mutation. *Cell*, **151**, 1431–1442.

21. Iulio,J. di, Bartha,I., Wong,E.H.M., Yu,H., Lavrenko,V., Yang,D., Jung,I., Hicks,M.A., Shah,N., Kirkness,E.F., *et al.* (2018) The human noncoding genome defined by genetic diversity. *Nature Genetics*, **50**, 333–337.
